# Emergent dolutegravir resistance in integrase-naïve, treatment experienced patients from Zimbabwe

**DOI:** 10.4102/sajhivmed.v23i1.1435

**Published:** 2022-10-26

**Authors:** Linda A. Mandikiyana Chirimuta, Margaret J. Pascoe, Sara Lowe

**Affiliations:** 1 Newlands Clinic, Ruedi Luethy Foundation, Harare, Zimbabwe

**Keywords:** HIV drug resistance, dolutegravir, integrase strand inhibitor, antiretroviral treatment, HIV

## Abstract

We report two cases of dolutegravir (DTG) resistance in highly treatment experienced patients. Monitoring for treatment failure and adherence support is important in highly treatment experienced patients taking DTG.

**What this study adds:**

Dolutegravir is the mainstay of HIV treatment programmes and emergence of drug resistance to DTG is of public health relevance.

## Introduction

The World Health Organization has recommended the inclusion of the integrase strand transferase inhibitor (InSTI), dolutegravir (DTG), as part of first-, second- and third-line antiretroviral treatment (ART) regimens. Dolutegravir is co-formulated with other antiretroviral medicines, well tolerated, has a high genetic barrier to resistance and is available in generic formulations. To date, few cases of emergent DTG resistance in integrase-naïve patients have been reported,^1^ but it is anticipated that with increasing use across treatment regimens, more cases will emerge, particularly in highly treatment experienced patients.

We describe two cases of highly treatment experienced, InSTI-naïve people living with HIV from Newlands Clinic, Harare, Zimbabwe, who developed InSTI drug-resistance mutations (DRMs) within 14 months of InSTI initiation.

## Ethical considerations

Written informed consent was obtained from the patient described in this case report. The patient gave his consent to have his clinical and demographic data to be used as well as his images.

Ethical approval to conduct a case report on these patients was obtained from the Medical Research Council of Zimbabwe. Clearance number: E/316.

## Patient presentation

### Case 1

An 18-year-old male patient was commenced on ART in September 2010, at seven years of age. His baseline CD4 count was 145 cells/μL. His first-line ART regimens were stavudine/lamivudine (3TC)/nevirapine, followed by stavudine/3TC/efavirenz (EFV), followed by zidovudine/3TC/EFV. Viral load (VL) results were not available until 2014, at which time he was noted to have virological treatment failure. An HIV genotypic resistance test done in January 2015 showed significant nucleoside reverse transcriptase inhibitor (NRTI) and non-nucleoside reverse transcriptase inhibitor (NNRTI) DRMs with no protease inhibitor resistance (see Table 1 for DRMs and sensitivity results). In January 2015 he was switched to second-line treatment with abacavir (ABC)/3TC/lopinavir/ritonavir (LPV/r), which was later simplified to ABC/3TC/Atazanavir/ritonavir (ATZ/r). Virological suppression was maintained for the duration of protease inhibitor therapy. In August 2019 he was switched to a single tablet regimen of tenofovir (TDF)/3TC/DTG; his VL was suppressed at the time of switch.

**TABLE 1:** Historical antiretroviral treatment regimens, treatment switch reasons, viral load results and genotype mutations.

Case	Duration/Date	ART regimen	Switch reason	VL at switch (copies/mL)	Genotype mutations	Stanford database interpretation
Case 1	September 2010 – January 2015	D4T/3TC/EFV AZT/3TC/EFV	Virological failure	70 098	**19 January 2015** **NNRTI:** A98G, K101E, V106M, Y181C, G190A **NRTI:** M184V, M41L, T215Y **PI:** none	EFV, NVP, ETR: High-level resistance 3TC, AZT: High-level resistance ABC: Intermediate resistance TDF: Low-level resistance
	January 2015 – August 2019	ABC/3TC/LPV/r ABC/3TC/ATZ/r	Treatment simplification	< 20		
	August 2019	TDF/3TC/DTG		< 20		
	February 2020	-		< 20		
	June 2021	-		47 530	**01 June 2021** **NNRTI:** A98G, K101E, V106M, Y181C, G190A **NRTI:** M184V, M41L, T215Y **PI:** none **InSTI:** E3138K, G140A, S147G, Q148R, N155H.	EFV, NVP, ETR: High-level resistance 3TC: High-level resistance ABC, TDF, AZT: Intermediate resistance DTG: High-level resistance
Case 2	June 2017 – June 2020	TDF/3TC/EFV	Virological failure	50 450		
	June 2020 – Aug 2020	ABC/3TC/ATZ/r	Treatment simplification	Unavailable		
	August 2020 – present	ABC/3TC/DTG		Unavailable		
	March 2021			6540		
	June 2021			841 269	**01 June 2021** **NNRTI:** L100I, K103N, E138G, V179E **NRTI:** K65R, Y115F, M184V **PI:** none **InSTI:** G118R	EFV, NVP: High-level resistance ETR: Intermediate resistance 3TC, TDF, ABC: High-level resistance AZT: Susceptible DTG: Intermediate resistance

ART, antiretroviral treatment; VL, viral load; D4T, stavudine; 3TC, lamivudine; EFV, efavirenz; AZT, zidovudine, ETR, etravirine; ABC, abacavir; TDF, tenofovir; NNRTI, non-nucleoside reverse transcriptase inhibitor; NRTI, nucleoside(tide) reverse transcriptase inhibitor; LPV, lopinavir; r, ritonavir; ATZ, atazanavir; DTG, dolutegravir; PI, protease inhibitor; InSTI, integrase strand transferase inhibitor.

Virological rebound occurred 11 months after initiation of the InSTI regimen. The patient reported a history of suboptimal adherence to TDF/3TC/DTG; no other risk factors for virological failure were identified. Despite adherence counselling his VL remained high, and an HIV genotypic resistance test taken 14 months after TDF/3TC/DTG initiation showed persistent NRTI and NNRTI DRMs, and extensive InSTI DRMs (see Table 1 for mutations and sensitivity analysis).

## Management and outcome

Following the genotypic resistance test, the patient was switched to TDF/3TC/boosted darunavir and adherence support was reinforced. Eight weeks after the switch, the VL was 400 copies/mL and at 24 weeks, the viral load was undetectable. He continues this regimen with 3-monthly VL monitoring and adherence support.

### Case 2

A 29-year-old female patient was diagnosed with HIV infection in June 2017 and commenced on TDF/3TC/EFV. Her baseline CD4 count and VL are unknown. Three years later, she was switched to ABC/3TC/ATZ/r following virological failure. Four weeks after treatment switch, she developed pulmonary tuberculosis and in error was given rifampicin with ATZ/r for two months. For treatment optimisation reasons, the ART regimen was switched to ABC/3TC/DTG, but DTG was dosed at 50 mg daily with rifampicin for four months instead of twice daily according to current guidelines. The VL at switch to ABC/3TC/DTG is unknown. The clinical response to treatment was poor and she was referred to Newlands Clinic. A VL taken after seven months on ABC/3TC/DTG was 6540 copies/mL, despite adherence counselling. A genotypic resistance test taken 10 months after initiation of DTG showed extensive NRTI DRMs and one major InSTI DRM (see Table 1 for mutations and sensitivity analysis). This patient admitted to inconsistent adherence both to tuberculosis treatment and ART.

## Management and outcome

The patient was retreated for tuberculosis with adherence monitoring and the clinical response was good with weight gain and symptom resolution. On completion of six months of tuberculosis therapy, her integrase resistance data was available, but the initial sequencing of reverse transcriptase and protease had failed and was being repeated. Due to the long history of poor adherence, it was assumed that backbone NRTI resistance was likely; therefore, a third-line regimen consisting of TDF/3TC, darunavir in addition to twice daily DTG was considered the most robust regimen. After receiving weekly enhanced adherence support her VL at week 12 was 98 copies/mL and at week 24 was 71 copies/mL.

## Discussion

We report two cases of treatment experienced, InSTI-naïve patients who developed InSTI resistance within 14 months of starting DTG. The first patient had significant NRTI resistance prior to switching to TDF/3TC/DTG and suboptimal adherence post treatment switch. The second patient also reported suboptimal adherence and may have had sub-therapeutic drug levels of DTG due to a drug interaction between DTG and rifampicin. Known risk factors for incident drug resistance include suboptimal medication adherence, drug interactions, a high baseline VL and active opportunistic infections.^1^ A similar case from South Africa has been described, of a treatment experienced patient who developed resistance to DTG after taking DTG 50 mg once a day with rifampicin, instead of the recommended twice daily dosing. Rifampicin is an inducer of the UGT1A1 and CYP3A4 pathways by which DTG is metabolised, resulting in reduced serum DTG concentrations. The current World Health Organization recommendation is to dose DTG at 50 mg 12-hourly for patients taking both medications concurrently.

Integrase strand transferase inhibitor resistance in those with previous exposure to first-generation InSTI has been well described; however, the development of InSTI resistance in patients taking DTG is rare in ART-naïve patients. In a systematic review, Cevik et al. report 15 cases of emergent InSTI resistance in patients on DTG: 5 cases in ART-naïve patients and 10 cases in ART experienced patients.^1^ As increasing numbers of cases of DTG resistance are reported, the question arises as to whether patients who are treatment experienced may be at higher risk of InSTI resistance. The use of DTG in patients with compromised NRTI backbones has raised concerns regarding the efficacy and durability of these regimens.^2^ However, emerging evidence from the NADIA, VISEND and DAWNING trials appears reassuring that viral suppression despite the presence of NRTI mutations is achievable.^3,4,5^ Despite the good virological suppression rates achieved in these trials, it is of concern that a small percentage of participants developed treatment emergent DTG resistance; 4.0% of NADIA partcipants at week 96 and 0.6% of DAWNING participants. Although the risk of InSTI resistance was modest in these clinical trials, it is likely to be increased in real-world settings particularly those with failing health systems.

### Conclusion

Despite the high barrier to resistance of second-generation InSTIs, emergent DRMs can occur in treatment experienced InSTI-naïve patients. Evaluation of background resistance, avoidance of drug interactions and adherence support could prevent the development of InSTI resistance.
